# Triggering Neurotransmitters Secretion from Single Cells by X-ray Nanobeam Irradiation

**DOI:** 10.1021/acs.nanolett.0c01046

**Published:** 2020-03-31

**Authors:** Federico Picollo, Giulia Tomagra, Valentina Bonino, Valentina Carabelli, Lorenzo Mino, Paolo Olivero, Alberto Pasquarelli, Marco Truccato

**Affiliations:** † Department of Physics, NIS Interdepartmental Centre, University of Torino and Italian Institute of Nuclear Physics, via Giuria 1, 10125 Torino, Italy; ‡ Department of Drug and Science Technology, NIS Interdepartmental Centre, University of Torino, Corso Raffaello 30, 10125 Torino, Italy; § Department of Chemistry, NIS Interdepartmental Centre, University of Torino, via Giuria 7, 10125 Torino, Italy; ∥ Institute of Electron Devices and Circuits, University of Ulm, 89069 Ulm, Germany

**Keywords:** X-ray synchrotron nanoirradiation, dopamine exocytosis, diamond microelectrode arrays, photocurrent detection, radiobiology

## Abstract

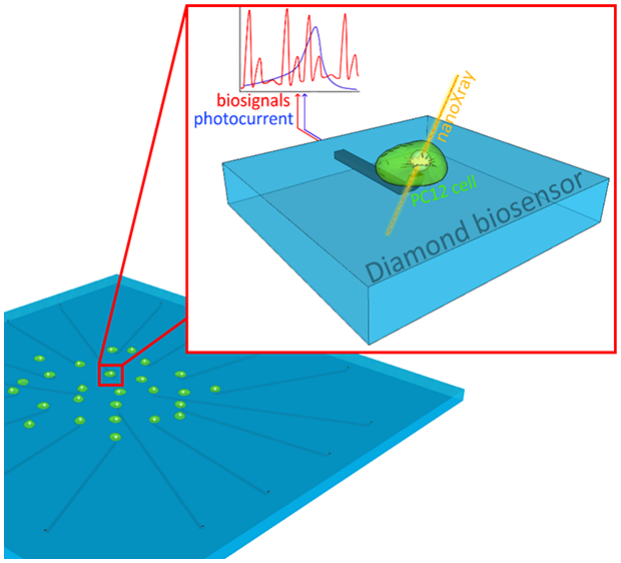

The employment of ionizing radiation is a powerful tool in cancer therapy, but beyond targeted effects, many studies have highlighted the relevance of its off-target consequences. An exhaustive understanding of the mechanisms underlying these effects is still missing, and no real-time data about signals released by cells during irradiation are presently available. We employed a synchrotron X-ray nanobeam to perform the first real-time simultaneous measurement of both X-ray irradiation and in vitro neurotransmitter release from individual adrenal phaeochromocytoma (PC12) cells plated over a diamond-based multielectrode array. We have demonstrated that, in specific conditions, X-rays can alter cell activity by promoting dopamine exocytosis, and such an effect is potentially very attractive for a more effective treatment of tumors.

Dopamine (DA) is an important monoamine neurotransmitter involved both in central nervous system regulation of cognition, behavior, mood, addiction, reward,^1−5^ and in multiple functional modulations of peripheral tissues and organs. The possible role of DA and its receptors in affecting the growth of some malignant tumors was hypothesized for the first time about 20 years after observing its large decrease in cancer tissues compared to normal ones.^6−8^ Nowadays, it has been proved that DA inhibits angiogenesis by affecting vascular permeability factor (VPF) and vascular endothelial growth factor (VEGF)-induced endothelial cell proliferation^9^ and that it reduces mesenchymal stem cell (MSC) and endothelial progenitor cell (EPC) migration.^10^ Increasing evidence indicates that DA plays an important role not only in mediating cross-talk between central nervous and immune systems^11,12^ but also at a peripheral level by acting on tumor-associated immunologic alterations.^13^ Indeed, DA is synthesized, with rare exceptions, in most types of immune cells^14^ and released to the extracellular environment after specific stimulation.^15^ Due to the increasing interest in the use of immunotherapy as an efficient tool to boost the occurrence of the abscopal effect^16,17^ and because of the DA interference with the immune system, a study of dopaminergic cell response to X-ray irradiation is necessary, especially in view of the potential synergy that it could have along with immunotherapy for malignant tumor treatment.

In this study, we have employed the PC12 immortalized cell line, which synthesizes DA and releases it upon membrane depolarization in a Ca^2+^-dependent way. The cellular exocytotic activity has been monitored by means of a device consisting of a single-crystal diamond substrate equipped with a multielectrode array of graphitic microchannels (μG-D-MEA). This device was fabricated out of high-quality artificial diamond substrate by means of a lithographic technique based on the use of MeV ions, which was optimized in previous studies (see Supporting Information for details). Its suitability to the fabrication of integrated cellular sensors for *in vitro* measurements has already been demonstrated in a series of previous works.^18−20^ As shown in Figure 1, each fabricated subsuperficial conductive microchannel is characterized by two emerging end-points, one in correspondence of the biological sample under investigation (i.e., the cells plated in the central region of the device) and the other at the input of the acquisition electronic chain (i.e., the readout contacts at the peripheral region).

**Figure 1:**
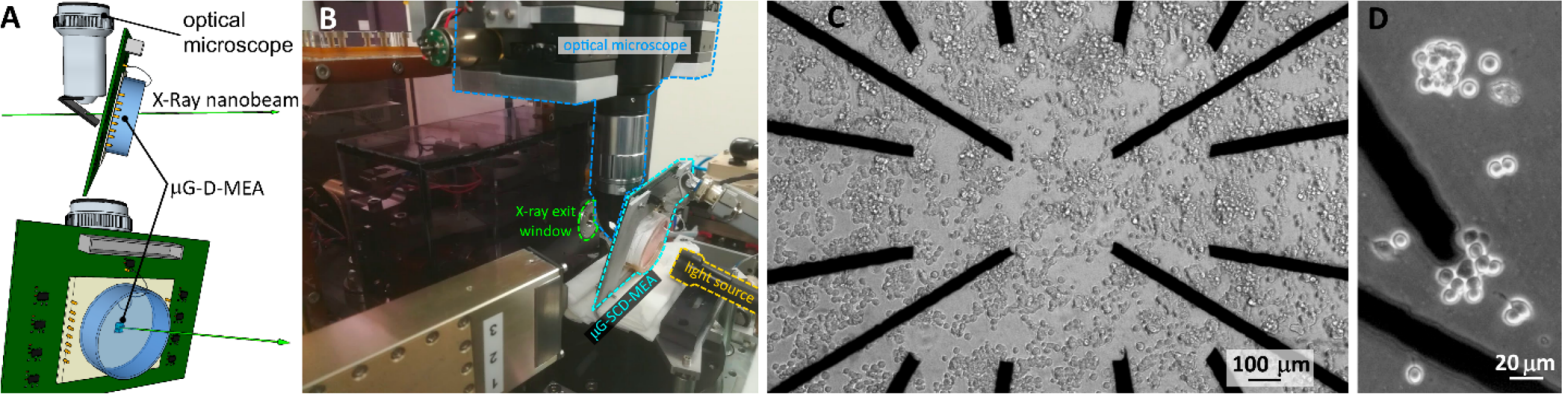
Single cell irradiation setup. Three-dimensional schematic (A) and picture (B) of the irradiation end-station. The X-ray nanobeam is focused on the vertically mounted diamond-based sensor. (C) Transmission micrograph of PC12 cells plated over a sensor. (D) Zoom of some cells close to a graphitic electrode.

Diamond biocompatibility allows plating cells directly over the surface of the sensor without altering the cellular activity over long-term measurements, as demonstrated in previous studies.^21^ As shown in Figure 1c, the graphitic microelectrodes are arranged in a 4 × 4 matrix over a surface of 0.4 mm^2^, and each of them is characterized by a 70 μm^2^ active region exposed to the surface of the substrate. This geometry allows sensing the exocytotic quantal release from individual cells by each of the 16 electrodes. A hermetic perfusion chamber equipped with an Ag/AgCl reference electrode immersed in the culturing medium completes the device. The wide electronic bandgap and the high carrier mobility of diamond enable the visible-light-blind detection of the incident X-ray nanobeam by means of the very same graphitic structures used for biosensing, which are employed in this case as photocurrent-sensing electrodes. Specifically, the different time-scales characterizing the signals induced by the incident ionizing radiation (i.e., ∼s) and the exocytotic signals from the cultured cells (i.e., ∼ms) allow sharing the same electronic chain, which therefore simultaneously records both types of signals in a single chronogram (Figures 2 and 4). The observation of the X-ray-induced photocurrent was employed during the cell exposition to define the irradiation starting time, providing a precise synchronization of the amperometric recording with cell irradiation.

**Figure 2:**
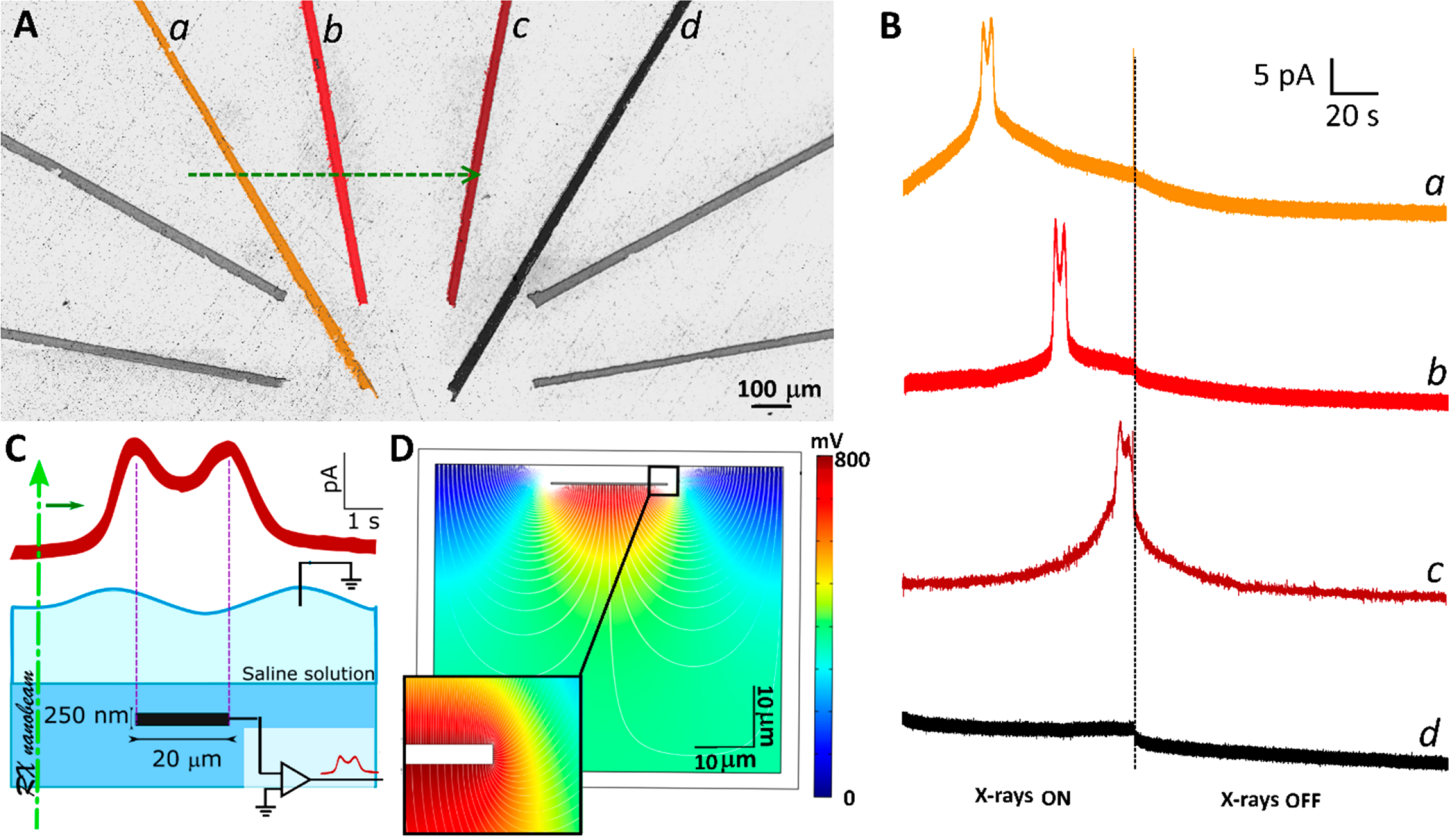
X-ray beam-induced current. (A) Optical micrograph of graphitic channels in which the path scanned by the X-ray nanobeam is highlighted by the green arrow. (B) Photocurrent signals generated by scanning the X-ray nanobeam across the sensor. The shape of the current spikes is associated with the beam position with respect to the graphitic channels. (C) Schematic of a channel cross-section with the corresponding electrical connections. (D) Color map of the electrostatic potential between the saline solution and one electrode (white rectangle), with a zoom in of an electrode corner, as determined from numerical simulations. The color scale ranges from 0 mV (blue) to 800 mV (red).

The experiment was performed at the ID16B beamline of the ESRF synchrotron facility, where a focused 17.4 keV X-ray beam with 55 nm × 60 nm spot size and high flux (up to ≈7 × 10^10^ photons s^–1^) is delivered in air. The sample was positioned in the focal plane of a conventional optical microscope, which was aligned to almost coincide with the focal plane of the X-ray beam^22^ (see Supporting Information for details). As shown in Figure 1a,b, the μG-D-MEA was placed vertically, with its back facing the incident X-rays. This configuration guarantees a negligible (i.e., < 9%) X-ray absorption before reaching the cultured cells, while avoiding any image distortion in optical imaging (Figure 1c,d). All of the measurements reported in this work were carried out by polarizing the 16 graphitic channels at +800 mV with respect to the physiological solution, which was grounded through a reference electrode.

Initially, the acquisition of the X-ray-induced signal was performed from a noncell-plated substrate. The beam was scanned along a line crossing three graphitic electrodes and stopped in correspondence of the third one (Figure 2a).

Signal recording started immediately after opening the shutter of the X-ray beam. The X-ray photocurrent signal is easily detectable as a significant increase of the recorded current (30–40 pA) with respect to the noise level (2–5 pA). This signal (Figure 2b) is characterized by a time pattern featuring a double peak in correspondence of the beam crossing each of the channels, which can be explained by considering the distribution of the electrostatic potential near the electrode boundaries, as shown by the finite element method (FEM) simulation reported in Figure 2d and Figure S2 of SI. As expected, the two main effects occurring after stopping the X-ray irradiation are (i) the disappearance of the photocurrent peaks, as demonstrated by the chronoamperogram collected from channel *c* (Figure 2b), and (ii) a slight reduction of the baseline for both irradiated (*c*) and not irradiated (*a, b, d*) channels. To correctly define the irradiation protocol for the subsequent experiments, the number of photons necessary to induce cell death was estimated. The cells were plated with ∼1 mM vital stain trypan blue, in order to selectively mark only the dead ones. The X-ray nanobeam was placed in correspondence of the center of each investigated cell, which was then exposed for increasing times until the color modification associated with the cell death was observed (Figure 3a). This protocol was repeated systematically by varying the X-ray flux in the 6 × 10^7^–7 × 10^10^ photons s^–1^ range, and the relevant data reported in Figure 3b are indicative of the inverse proportionality between the cell survival time and the photon flux. The minimum number of photons necessary to induce cell death was evaluated as (4.0 ± 1.8) × 10^10^ from the product of the survival time by the photon flux.

**Figure 3:**
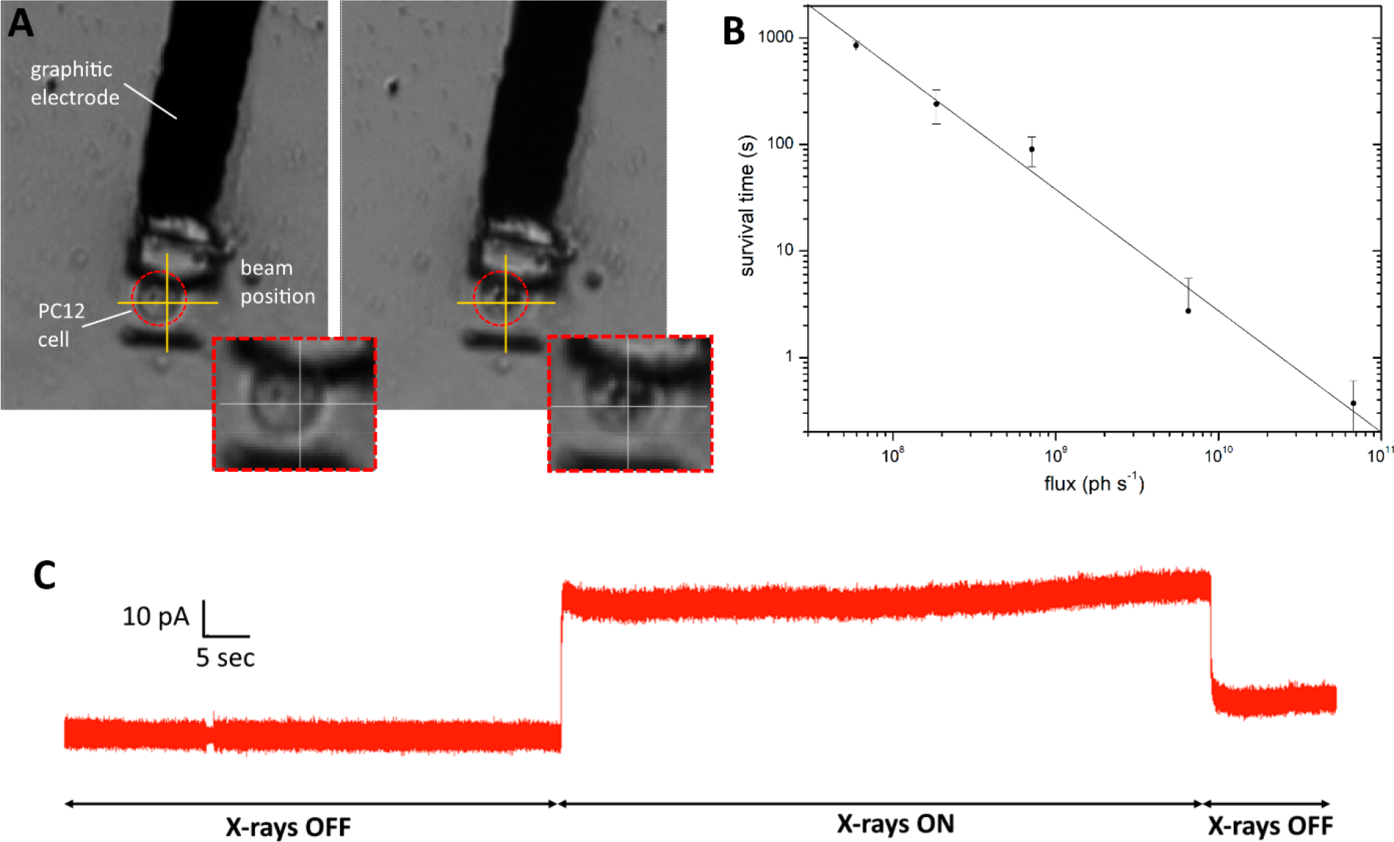
Evaluation of the X-ray nanobeam lethal dose. (A) Micrograph of a PC12 cell placed close to a graphitic microelectrode before (left) and after (right) the delivery of an X-ray lethal dose. The zoom-ins reported in the red boxes highlight the changes in the cell color associated with the presence of trypan blue vital stain. (B) Survival time vs X-ray flux. The unitary negative slope of the data in log–log scale is indicative of a linear trend. (C) The chronoamperogram, recorded during higher photon flux irradiation, shows the increase of the signal baseline associated with the detection of an X-ray photocurrent. No signals associated with the deterioration of the cell membrane are visible.

Amperometric measurements were performed during the exposure of inactive cells to the above-defined lethal dose in order to check for the possible presence of spurious cellular signals occurring in correspondence of cell death and associated with the increase of permeability or deterioration of the cellular membrane. These irradiations were performed at the maximum photon flux (7 × 10^10^ photons s^–1^) for ∼75 s, obtaining chronoamperograms of the exocytotic activity (see Figure 3c). In these chronoamperograms, only a ∼30 pA increase of the signal baseline associated with the detection of photocurrent can be observed, thus confirming the absence of the above-mentioned undesired signals that could have been erroneously attributed to exocytotic spikes.

Other irradiations were performed at a flux of 7 × 10^8^ photons s^–1^, thus ensuring to keep the cells alive for at least a few tens of seconds. Only cells placed directly to the graphite electrode were selected, allowing the optimal detection of exocytotic signals. The chronoamperogram reported in Figure 4a shows the activation of an intense exocytotic pattern after starting the raster-scanning of the X-ray beam across a single cell that was initially inactive. In this case, many amperometric spikes overlap with the broad and intense peaks associated with the detection of the photocurrent. The photocurrent signals are characterized by a slow rise ascribable to the direct irradiation of the electrode and a sharp decay when the beam moves further and impinges the insulating diamond surrounding the graphite electrode. On the other hand, the parameters associated with the kinetics (i.e., full width half-maximum *t*_1/2_) and intensity (i.e., maximum peak current *I*_max_ and quantal charge *Q*) of the exocytotic spikes detected from irradiated cells are statistically consistent with the control data set (*p* > 0.05, ANOVA followed by Bonferroni post hoc comparison, Figure 4b,c), while DA release frequency presents a significant increment only during the irradiation.

**Figure 4:**
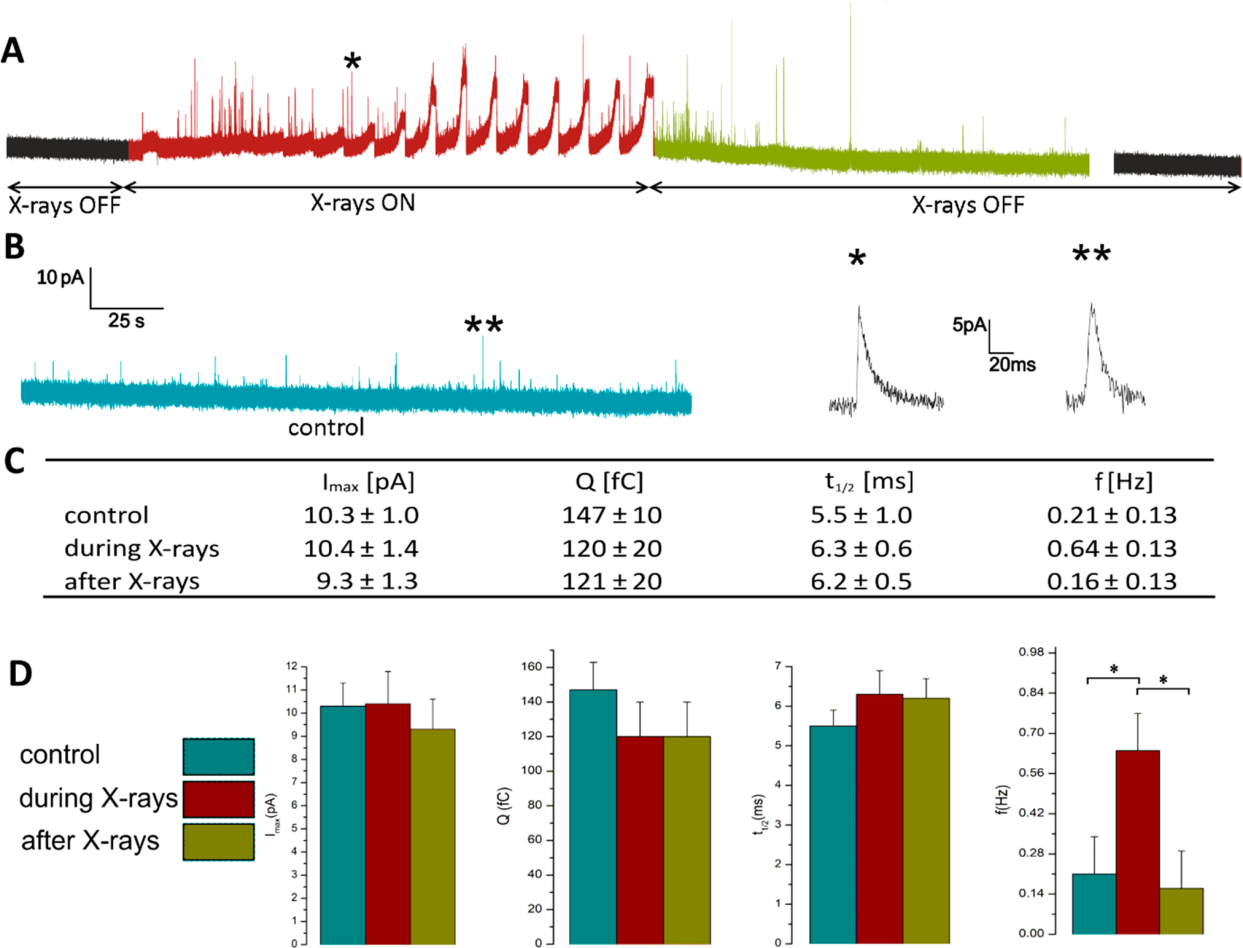
X-ray stimulated neurotransmitters release. (A) Amperometric recording of X-ray-induced exocytosis with zoom-in of a spike (labeled as *). The switching-on and off of the beam is highlighted. (B) Typical chronoamperogram of spontaneous exocytotic events from PC12 cells with zoom-in of a spike (labeled as **). (C) Table reporting the characteristic parameters of the exocytotic events for both X-ray stimulated and spontaneous events. (D) Histograms illustrating the data reported in (C).

Control amperometric recordings of the spontaneous exocytosis were acquired by adopting the same measurement protocol but without exposing the cells to X-ray radiation. The invariance of the above-mentioned parameters demonstrates that in the reported conditions, the X-ray irradiation does not affect the exocytotic pathway, while the appearance of frequent exocytotic events is an unequivocal and direct evidence that X-ray irradiation can have a stimulation effect of the exocytotic activity.

In principle, the radiation-induced stimulation of DA release could be ascribed to two different phenomena, that is, the increase of the cell temperature^23,24^ and/or the generation of free radicals.^25^ Concerning the first hypothesis, several studies have shown that a temperature increase promotes the exocytosis, affecting voltage-gated ion channels, ion pumps, and temperature-gated channels. For example, L-type calcium channels can be depolarized at temperatures higher than 39 °C.^24^

To explore this first hypothesis, a simulation of the temperature profile induced upon irradiation of the cell was performed, by solving with the finite element method software COMSOL Multiphysics the Fourier heat equation for our experiment. To this purpose, an overestimation of the heating process was introduced by assuming that all the power delivered by the X-ray source is converted into heat without contribution from the environment since the experimental hutch temperature is kept constant at a tent of a degree. The space distribution of the power density was defined by the attenuation length of the impinged materials and by the Gaussian profile of the X-ray beam. Finally, in order to consider the time pattern of the synchrotron radiation, the source was also modulated in time. The temperature evolution of the cell, after the first X-ray pulses of irradiation, is shown in Figure 5. Also in virtue of the very high thermal conductivity of the diamond substrate, the very modest temperature increase does not justify any thermal stimulation of the cell.

**Figure 5:**
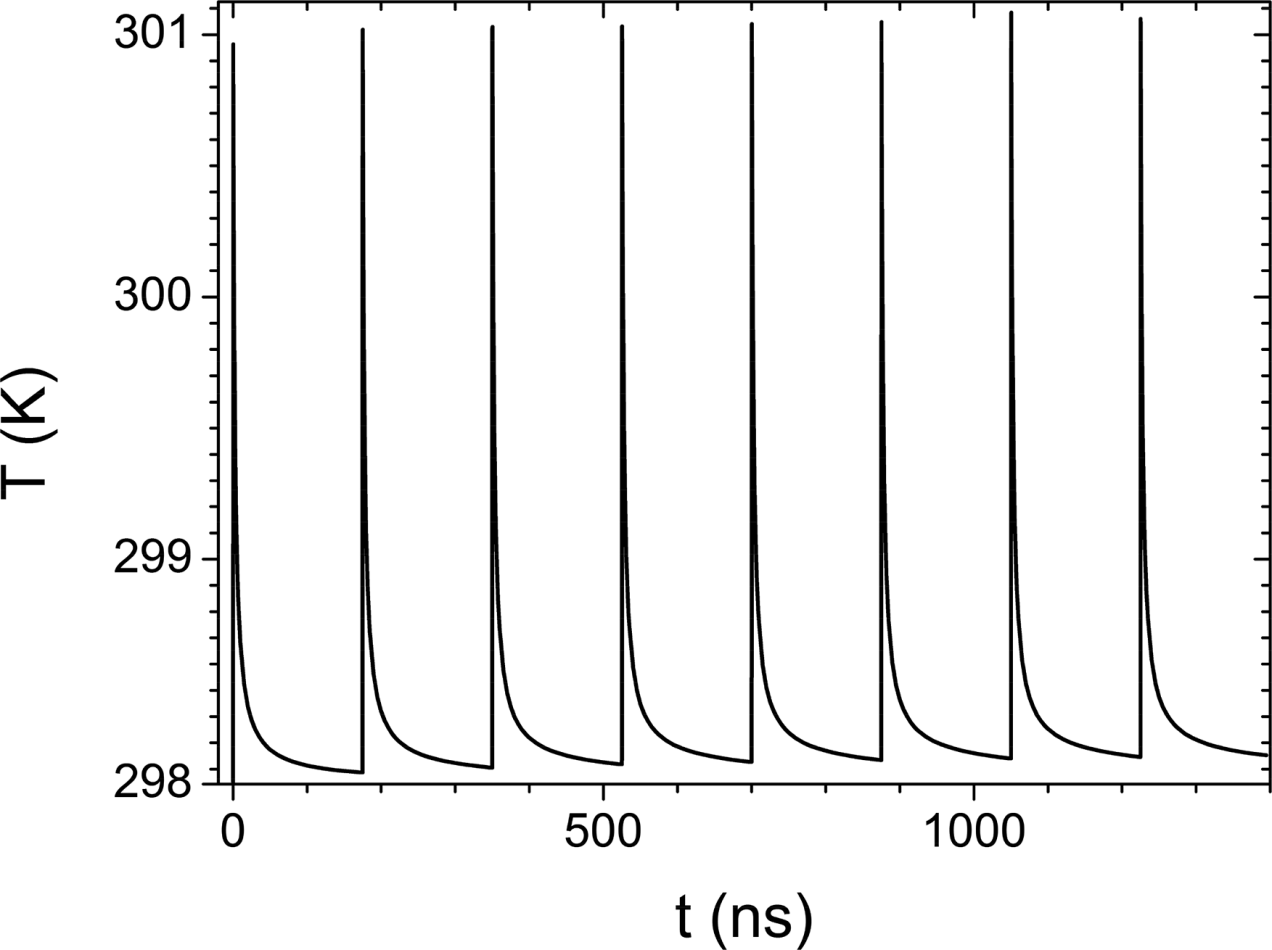
Temperature evolution of the cell calculated at the point of maximum temperature. The temperature modulation is generated by the time pattern of the synchrotron source. The initial temperature of the system was set equal to ambient temperature (298.15 K).

The second hypothesis concerns the effects of radiation-induced free radicals; indeed, it has been proved that hydrogen peroxide radicals induce an increase in the cytosolic Ca^2+^ concentration, thus stimulating exocytic release.^25,26^ On this aspect, further tests will be necessary for an unambiguous identification of the causes of the observed phenomena.

The results presented here demonstrate the high potentiality of the innovative methodology based on the combined use of diamond-based integrated biosensing/detector devices and of nanometric X-ray beams from synchrotron sources, which enables the study of the effects of ionizing radiation at the individual cell level by recording their activity in real-time while simultaneously monitoring the delivered beam. This approach discloses new perspectives in radiobiological experiments from the point of view of the investigation of radiation effects on specific organelles with submicrometric spatial resolution. Finally, we have also shown that, under some conditions, X-rays stimulate DA release, which is a novel effect and could potentially have great implications for radiotherapy of malignant tumors.

## Supporting Information Available

Fabrication processes, technical specifications, and characterization of the μG-D-MEAs; FEM simulations of the photocurrent detection and X-ray-induced temperature variation; PC12 cell line description and culture protocol; amperometric spikes description and analysis; ID16B-NA ESRF beamline (PDF)

## Abbreviations

μG-D-MEA: multielectrode array of graphitic microchannels
DA: dopamine
VPF: vascular permeability factor
VEGF: vascular endothelial growth factor
MSC: mesenchymal stem cell
EPC: endothelial progenitor cell
FEM: finite element method
