# Supplementary material for: Triggering Neurotransmitters Secretion from Single Cells by X-ray Nanobeam Irradiation
Source: Nano Lett. 2020 Mar 31;20(5):3889–94. doi: 10.1021/acs.nanolett.0c01046 (PMC7997629; doi:10.1021/acs.nanolett.0c01046)
Supplement: Supplementary file 1 — nl0c01046_si_001.pdf [file nl0c01046_si_001.pdf]

Triggering neurotransmitters secretion from single cells by x-ray nanobeam irradiation

Federico Picollo[1], Giulia Tomagra[2], Valentina Bonino[1], Valentina Carabelli[2], Lorenzo Mino[3], Paolo Olivero[1], Alberto Pasquarelli[4], Marco Truccato[1]*

[1] Department of Physics, NIS Inter-departmental Centre, University of Torino, and Italian Institute of Nuclear Physics, via Giuria 1, 10125 Torino, Italy

[2] Department of Drug and Science Technology, NIS inter-departmental centre, University of Torino, Corso Raffaello 30, 10125 Torino, Italy

[3] Department of Chemistry, NIS Inter-departmental Centre, University of Torino, via Giuria 7, 10125 Torino, Italy

[4] Institute of Electron Devices and Circuits, University of Ulm, 89069 Ulm, Germany

SUPPORTING INFORMATION

Materials and Methods

μG-D-MEAs – fabrication processes

Multi-electrode arrays of sub-superficial graphitic channels (μG-D-MEAs) were fabricated in high-quality single-crystal diamond substrates produced by ElementSix by Chemical Vapor Deposition. The samples were 4.5×4.5×0.5 mm^3 in size, cut along the (100) crystalline direction

and optically polished on the two opposite large faces. The crystals were classified as "type IIa", being characterized by nominal concentrations of substitutional nitrogen and boron lower than 1 ppm and 0.05 ppm, respectively.

As extensively detailed in previous works [1,2], MeV ion beam lithography allows the creation of conductive graphitic paths embedded in the surrounding diamond crystal matrix (1.8 MeV He beam delivering a fluence of 1×10^{17} cm^{-2}). After a high-temperature thermal annealing treatment (950 °C in high vacuum for 2 hours), the phase transition of the diamond crystal to graphite is promoted in the regions damaged with a vacancy density above the critical value of 3×10^{22} cm^{-3} (usually referred to as "graphitization threshold")[3–5].

The possibility of controlling the formation of the graphitic structures with high resolution in the three spatial dimensions was obtained by the employment of a specific set of implantation masks. A 100 μm thick stencil metal mask allowed collimating the ion beam, thus defining the lateral dimensions of the created structures. Complementarily, a copper mask with thickness varying in the 0-5 μm range was employed to modulate the ion energy and thus their penetration depth in the sample, ultimately resulting in the fabrication of graphitic micro-channels emerging at the surface of the substrate in correspondence of the desired locations.

<u>μG-D-MEAs – technical specifications</u>

The employed biosensors consist of 16 channel multi-electrodes arrays, which allowed the collection of independent and simultaneous amperometric signals from different cultured cells[6–8]. The chip carrier was designed to perform *in vitro* measurements on cells directly cultured on the diamond surface and was therefore equipped with a 60 ml hermetic culture chamber with a Ag/AgCl reference electrode directly mounted in the cap.

The front-end electronics are based on low-noise transimpedance amplifiers, having an input bias current of 1 pA and the gain set by feedback-resistors of 100 MΩ. The signals were converted by a USB-6216 ADC module (National Instruments) equipped with Bessel low-pass filters of the sixth order with a bandwidth of 5 kHz. This configuration was optimized for amperometric acquisitions with sampling rate up to 25 kHz. The bias applied to the channels was +800 mV, a value that guarantees the oxidation of the dopamine with an optimal signal-to-noise ratio.

μG-D-MEAs – characterization

Cyclic voltammetry plots of four representative channels performed in saline solutions (Tyrode) without and with 75 μM dopamine are reported in figure S1. The measurements were performed by sweeping the electrode bias between -0.5 V and +1.2 V, with a scan rate of 10 mV s^{-1}. From the literature, the oxidation of dopamine molecules occurs in the range +0.6 V ÷ +0.8 V [9]. By analysing the collected curves from the solution enriched with dopamine with respect to the reference one, it was possible to identify that the bias which optimizes the signal-to-noise ratio is ~800 mV.

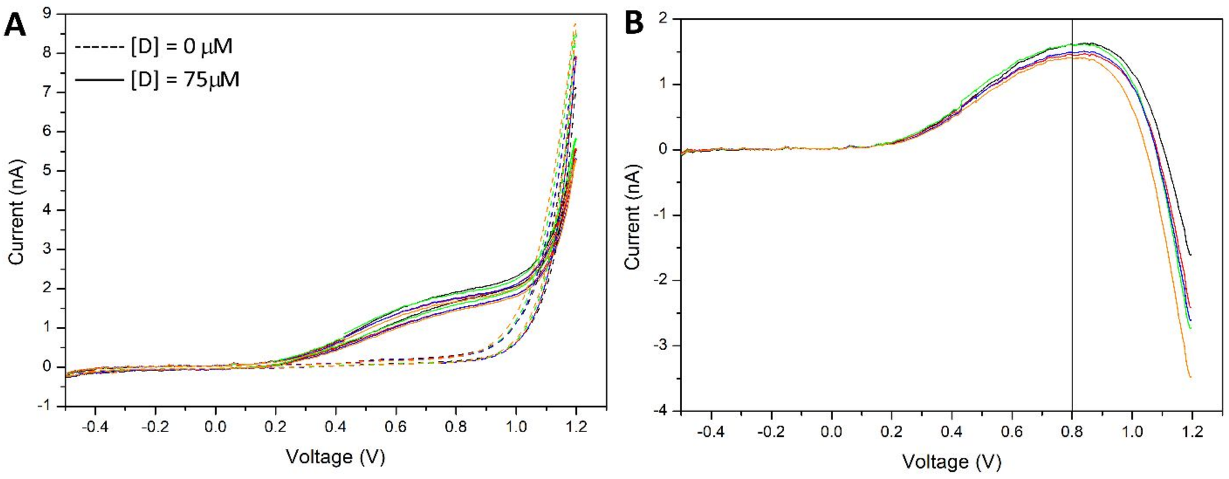

Figure S1. A) Cyclic voltammetry recorded from four representative channels from a saline solution without and with the addition of dopamine (75 μM). B) Curve obtained by the subtraction of the rising branch of cyclic voltammetry recorded from solutions with [D] = 75 μM with those without dopamine for all the four representative channels.

FEM simulations – photocurrent detection

The electrostatics of the device was calculated by solving the 2D Laplace equation relevant to the geometry sketched in Fig. 2c with a FEM software (COMSOL Multiphysics). Fig. 2d shows the relevant electrostatic potential map with the electric field streamline plot, which highlights the increase of the electric field in correspondence of the electrode boundaries, reproducing the experimental data (see Fig. 2 of Supporting Information).

The current generated by X-ray absorption was calculated by solving the continuity equations of the carriers, which provide the carrier concentration distributions and therefore the current collected at the buried and top electrodes. The carrier mobilities were extracted from Ref. [10]. The carrier lifetimes were set equal to a constant value in the bulk material (τ_e = 0.4 ns and τ_h = 1 ns for electrons and holes, respectively), whereas in the cap layer overlying the graphitic channel we assumed an exponential variation of the above-mentioned values (degrading from the top surface

to the electrode), which reproduces the inverse of the damage (vacancy profile) evaluated by SRIM2013 simulations[11]. The source term was represented by a Gaussian horizontal profile which the amplitude was exponentially decreasing with an attenuation coefficient of 2.86 μm^{-1} [12].

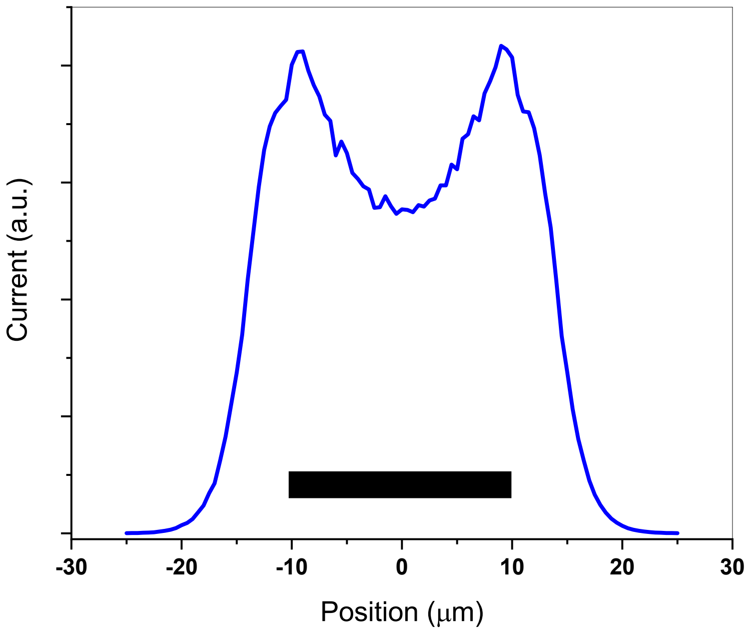

Figure S2. Simulated profile of the photocurrent collected at the buried electrode. The black rectangle represents the position of one of the electrodes. The simulated curve qualitatively resembles the experimental profile shown in Figure 2

FEM simulations – X-ray induced temperature variation

The simulation of the temperature increase induced in the cell by X-ray irradiation was simulated solving the following heat conduction problem with a FEM software (COMSOL Multiphysics):

$$\rho C_p \frac{\partial T}{\partial t} + \nabla \cdot (-k\nabla T) = Q$$

where T represents the temperature field, ρ is the material density, Cp is the heat capacity at constant pressure, k is the thermal conductivity, and Q represents the heat source. The dissipation power exerted by the convective motion of liquids was disregarded. The thermal parameter of the cell were assumed equal to the typical one for adrenal gland tissue [13]. The geometrical size of the domains adjacent to the cell were determined in order to not influence the simulation results. At the model external boundaries a temperature of 295.15 K was fixed.

PC12 cell line

PC12 cell line derives by pheochromocytoma of the rat adrenal medulla and is immortalized in order to proliferate indefinitely. This cell line is very well characterized and therefore adopted in several research works [14–16]. It is satisfactorily homogeneous with genetically identical populations, which aid in providing consistent and reproducible results. This cell line presents exocytosis mediated by vesicles containing catecholamines, mostly dopamine and a limited amount of norepinephrine [17].

PC12 cell culture

PC12 cells were maintained in RPMI (Invitrogen) containing 10% horse serum (Invitrogen), 5% fetal bovine serum (Invitrogen) and 2% antibiotic/antimitotic (pen/strep Invitrogen). Cells were treated with trypsine-EDTA (0.25% Sigma-Aldrich) to remove cells by flasks. All diamond chips were coated with collagen type I (Sigma-Aldrich). The μG-D-MEAs were coated for 1 hour with collagen, which was then removed with a gentle wash of Phosphate-buffered saline (PBS). Cell cultures were maintained at a temperature of 37 °C in a 5% CO_2 atmosphere. The experiments were performed 24 hours after culturing.

Amperometric spikes

Amperometric spikes present a characteristic shape ascribable to the kinetic of the fusion of the vesicles with the cell membrane during the exocytosis, that can be described with the following steps:

1) docking and priming of the vesicles mediated by the SNAREs proteins;
2) the fusion of the vesicles with the cell membrane starts with the formation of a nanometric fusion pore,
3) the pore expands releasing (usually completely) the vesicle content in the extracellular medium.

Figure S3 correlates steps to the amperometric current signal recorded when a cell is in contact with the microelectrode.

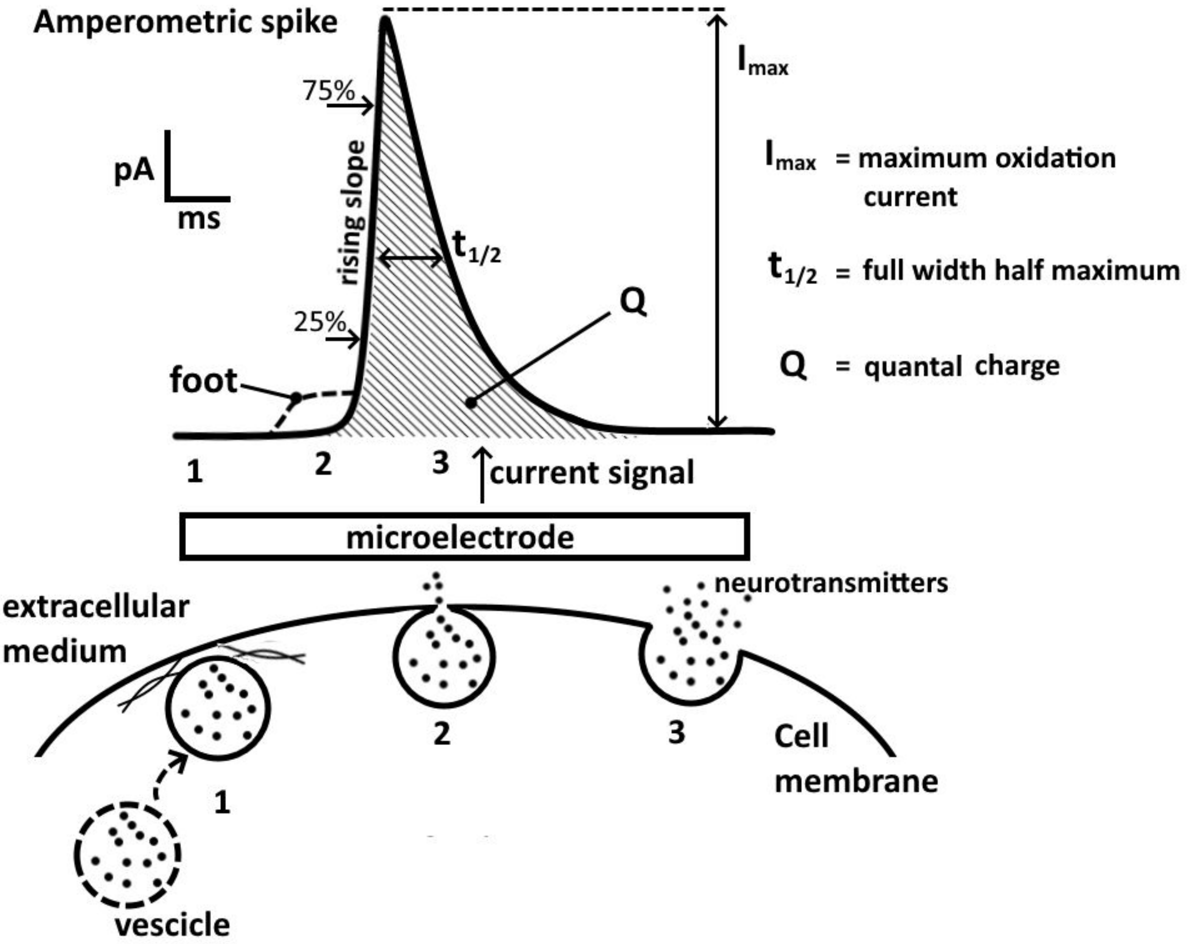

Figure S3. Electrical signal shape and parameters of the current spike generated by a quantal release event. Different steps of the exocitotic process are represented in the bottom of the figure.

Spikes analysis

Spikes analysis was performed using the "Quanta Analysis" routine of Igor pro software by waveMetrics. During the data analysis, diffusional events represent a source of uncertainty because the random distance of the cell from the electrode affects the secretion parameters, therefore a cut-off on the rise time parameter is applied to post-select only the spikes arising

from the cells that are placed in direct contact with the electrod. Signals characterized by a rise time larger than 3 ms (measured from 25% to 75% of the rising slope, see figure S1) are removed from the analysed pool."

ID16B-NA ESRF beamline

Irradiation experiments were carried out at the ID16B Nano-Analysis beamline of the European Synchrotron Radiation Facility (ESRF) in Grenoble, France. This beamline is equipped with primary optics consisting of a double white beam mirror and a double crystal monochromator (not employed in our experiment) located respectively at 30 m and 35 m from the in-vacuum undulator, and with focusing optics placed 165 m from the undulator to achieve a high degree of demagnification. The focusing optics system is represented by a pair of elliptical-shaped, multilayer-coated Kirkpatrick-Baez (KB) mirrors with a beam divergence of 3 mrad, whereas an optical microscope and a piezoelectric positioning stage are available to align the sample with the beam. The focal points of the microscope and of the KB mirrors coincide within experimental error when the optical path is in air. The FWHM beam sizes measured in these conditions by means of the knife-edge method were 55 nm (horizontal) × 60 nm (vertical). However, due to experimental geometry, the X-ray nano-beam crossed the whole 500 μm thickness of the diamond substrate before delivering the dose to the cells during irradiation. The storage ring was operated in the 16 bunch top-up mode and we selected the so-called "pink mode" (i.e. $\Delta E/E \approx 10^{-2}$) for the beam in order to reach the maximum possible flux of 6.7×10^{10} photons s^{-1} at the cell position. Si and Al attenuators with different thickness were employed to decrease the flux down to a possible minimum value of 6.4×10^{6} photons s^{-1}.

REFERENCES

(1) Picollo, F.; Gatto Monticone, D.; Olivero, P.; Fairchild, B. a.; Rubanov, S.; Prawer, S.; Vittone, E. Fabrication and Electrical Characterization of Three-Dimensional Graphitic Microchannels in Single Crystal Diamond. *New J. Phys.* **2012**, *14* (5), 053011.

(2) Picollo, F.; Olivero, P.; Bellotti, F.; Pastuović, Ž.; Skukan, N.; Lo Giudice, A.; Amato, G.; Jakšić, M.; Vittone, E. Formation of Buried Conductive Micro-Channels in Single Crystal Diamond with MeV C and He Implantation. *Diam. Relat. Mater.* **2010**, *19* (5–6), 466–469.

(3) Gippius, A.; Khmelnitskiy, R. A. R.; Dravin, V. A.; Tkachenko, S. D. Formation and Characterization of Graphitized Layers in Ion-Implanted Diamond. *Diam. Relat. Mater.* **1999**, *8* (8–9), 1631–1634.

(4) Battiato, A.; Lorusso, M.; Bernardi, E.; Picollo, F.; Bosia, F.; Ugues, D.; Zelferino, A.; Damin, A.; Baima, J.; Pugno, N. M.; et al. Softening the Ultra-Stiff: Controlled Variation of Young's Modulus in Single-Crystal Diamond by Ion Implantation. *Acta Mater.* **2016**, *116*, 95–103.

(5) Khmelnitsky, R. A.; Dravin, V. A.; Tal, A. A.; Zavedeev, E. V.; Khomich, A. A.; Khomich, A. V.; Alekseev, A. A.; Terentiev, S. A. Damage Accumulation in Diamond during Ion Implantation. *J. Mater. Res.* **2015**, *30* (09), 1583–1592.

(6) Picollo, F.; Battiato, A.; Bernardi, E.; Plaitano, M.; Franchino, C.; Gosso, S.; Pasquarelli, A.; Carbone, E.; Olivero, P.; Carabelli, V. All-Carbon Multi-Electrode Array for Real-Time in Vitro Measurements of Oxidizable Neurotransmitters. *Sci. Rep.* **2016**, *6*, 20682.

(7) Picollo, F.; Battiato, A.; Bernardi, E.; Marcantoni, A.; Pasquarelli, A.; Carbone, E.; Olivero,

P.; Carabelli, V. Microelectrode Arrays of Diamond-Insulated Graphitic Channels for Real-Time Detection of Exocytotic Events from Cultured Chromaffin Cells and Slices of Adrenal Glands. *Anal. Chem.* **2016**, *88* (15), 7493–7499.

(8) Carabelli, V.; Marcantoni, A.; Picollo, F.; Battiato, A.; Bernardi, E.; Pasquarelli, A.; Olivero, P.; Carbone, E. Planar Diamond-Based Multiarrays to Monitor Neurotransmitter Release and Action Potential Firing: New Perspectives in Cellular Neuroscience. *ACS Chem. Neurosci.* **2017**, *8* (2), 252–264.

(9) Venton, B. J.; Wightman, R. M. Psychoanalytical Electrochemistry: Dopamine and Behavior. *Anal. Chem.* **2003**, *75* (19), 414 A-421 A.

(10) Pernegger, H.; Roe, S.; Weilhammer, P.; Eremin, V.; Frais-Kölbl, H.; Griesmayer, E.; Kagan, H.; Schnetzer, S.; Stone, R.; Trischuk, W.; et al. Charge-Carrier Properties in Synthetic Single-Crystal Diamond Measured with the Transient-Current Technique. *J. Appl. Phys.* **2005**, *97* (7).

(11) Ziegler, J. F.; Ziegler, M. D.; Biersack, J. P. SRIM - The Stopping and Range of Ions in Matter (2010). *Nucl. Instruments Methods Phys. Res. Sect. B Beam Interact. with Mater. Atoms* **2010**, *268* (11–12), 1818–1823.

(12) Price-Whelan, A.; Poon, C. K.; Benson, M. A.; Eidem, T. T.; Roux, C. M.; Boyd, J. M.; Dunman, P. M.; Torres, V. J.; Krulwich, T. A. Tanscriptional Profiling of Staphylococcus Aureus during Growth in 2 M NaCl Leads to Clarification of Physiological Roles for Kdp and Ktr K+ Uptake Systems. *MBio* **2013**, *4* (4), 116.

(13) McIntosh, R. L.; Anderson, V. A Comprehensive Tissue Properties Database Provided for

the Thermal Assessment of a Human at Rest. *Biophys. Rev. Lett.* **2010**, *5* (3), 129–151.

(14) Kozminski, K. D.; Gutman, D. A.; Davila, V.; Sulzer, D.; Ewing, A. G. Voltammetric and Pharmacological Characterization of Dopamine Release from Single Exocytotic Events at Rat Pheochromocytoma (PC12) Cells. *Anal. Chem.* **1998**.

(15) Westerink, R. H. S.; Ewing, A. G. The PC12 Cell as Model for Neurosecretion. *Acta Physiol.* **2008**, *192* (2), 273–285.

(16) Chen, T. K.; Luo, G.; Ewing, A. G. Amperometric Monitoring of Stimulated Catecholamine Release from Rat Pheochromocytoma (PC12) Cells at the Zeptomole Level. *Anal. Chem.* **1994**, *66* (19), 3031–3035.

(17) Carter, M.; Shieh, J. *Guide to Research Techniques in Neuroscience*; Elsevier, 2015.
